# Assessment of the systemic immune‐inflammation index in type 2 diabetic patients with and without dry eye disease: A case‐control study

**DOI:** 10.1002/hsr2.1954

**Published:** 2024-05-01

**Authors:** Amani Y. Alhalwani, Shatha Jambi, Anwar Borai, Muhammad Anwar Khan, Hashem Almarzouki, Mohieldin Elsayid, Abdullah Fahad Aseri, Nada O. Taher, Ali Alghamdi, Abdulwahab Alshehri

**Affiliations:** ^1^ College of Science and Health Professions King Saud bin Abdulaziz University for Health Sciences Jeddah Saudi Arabia; ^2^ Department of Biomedical Research King Abdullah International Medical Research Center Jeddah Saudi Arabia; ^3^ King Abdulaziz Medical City Jeddah Saudi Arabia; ^4^ Faculty of Medicine King Abdulaziz University Jeddah Saudi Arabia; ^5^ College of Medicine Almaarefa University Riyadh Saudi Arabia

**Keywords:** dry eye disease (DED), neutrophil‐to‐lymphocyte ratio (NLR), platelet‐to‐lymphocyte ratio (PLR), systemic immune‐inflammation index (SII), type 2 diabetes (DM2), type 2 diabetes with dry eye disease (DM2‐DED)

## Abstract

**Background:**

The inflammation plays a role in the pathophysiology of type‐2 diabetes progression, and the mechanism remains unclear. The systemic immune‐inflammation index (SII) is a novel inflammatory marker for type 2 diabetes patients and integrates multiple indicators in complete blood counts and routine blood tests.

**Aim:**

Since there is no international diagnostic standard for dry eye disease (DED), this study uses low‐cost inflammatory blood biomarkers to investigate the correlation between SII and DM2–DED and determine the diagnosis indices of other biomarkers in DM2–DED.

**Methodology:**

A case‐control retrospective analysis of totel patients *n* = 293 randomly selected and categorized into four groups: DED, DM2, DM2‐DED, and healthy subjects. Demographic and blood biomarker variables were classified as categorical and continuous variables. The platelet‐to‐lymphocyte ratio (PLR), lymphocytes‐to‐lymphocyte ratio, neutrophil‐to‐lymphocyte ratio (NLR), and SII were calculated platelet count multiply by NLR and analyzed for their correlation for all groups.

**Results:**

Focusing on DM2‐DED patients was more common in females, 59.6%, than in males, 40.2%. The mean ages were 60.7 ± 11.85 years, a statistically significant difference with all groups. In the study group DM2‐DED, there was an increase in all blood markers compared to all remaining groups except PLR. Only neutrophil, hemoglobin A1c (HbA1c), and fasting blood sugar levels were statistically significant differences in DM2‐DED patients (*p* > 0.001, *p* < 0.001, and *p* < 0.001, respectively) compared to all groups. There was a positive correlation between HbA1c and PLR, HbA1c and NLR, and HbA1c and SII (*r* = 0.037, *p* = 0.705; *r* = 0.031, *p* = 0.754; and *r* = 0.066, *p* < 0.501, respectively) in the DM2‐DED group.

**Conclusion:**

This study demonstrated that elevated SII values were linked to elevated HbA1c in DM2‐DED patients. The potential of SII and HbA1c as early diagnostic indicators for ocular problems associated with diabetes mellitus is highlighted by their favorable connection in diagnosing DM2‐DED.

## 1 INTRODUCTION

Dry eye disease (DED) is a common inflammatory disorder of the tear film among adults worldwide. It frequently arises in conjunction with other illnesses, as a result of environmental stimuli, or even due to the use of some medications, such as antihistamines, which are available over the counter. It can also occur due to ocular surgery, computer screen exposure, or the use of contact lenses.^1^

DM has long been linked to an increased risk of the development of chronic ocular disorders such as DED.^2^, ^3^ The prevalence of DED is 15%−33% in diabetic patients over 65 years of age and is 50% more common in women than men.^4^ In patients with DM, decreased corneal sensitivity and poor reflex‐induced tear secretion may also contribute to developing DED. Additionally, oxidative stress induces by diabetes which contribute in developping DED. Previous studies have examined the relationship. In a hospital‐based study, 54% of people with diabetes had DED, and a statistically significant difference correlation was found between the durations of DM and DED.^5^ Eventually, uncontrolled DM with DED (DM‐DED) causes visual impairment and corneal erosions, leading to secondary microbial infections.^6^ DED is frequently associated with a disruption of the corneal epithelium, which, in turn, opens the door to microbial invasion and can lead to microbial keratitis.^7^, ^8^ To our knowledge, the degree to which DM may increase the chance of developing DED is unknown.

Blood biomarkers are used to detect microbial invasions. An improved understanding of the blood biomarkers associated with DM2‐DED and their influence on the disease pathophysiology would aid in the early detection, diagnosis, and management of the disease. DED is associated with raised levels of glycated hemoglobin A1c (HbA1c).^9^ Inflammation and immunity have both been implicated in the pathogenesis of DED. Hyperglycemia triggers an inflammatory cascade in the lacrimal functional unit, which then triggers both innate and adaptive immune responses.^10^ Nevertheless, few studies have been performed to investigate the exact mechanisms that underlie the onset of diabetes‐induced DED, the associated risk factors, or hematological changes associated with inflammatory mediators that could be used as biomarkers.^1^

The levels of production of inflammatory cytokines such as interleukin (IL)‐1α, IL‐1β, tumor necrosis factor‐A, and matrix metalloproteinase‐9 have been implicated in the pathophysiology of DED.^11^ Also, levels of IL‐6, IL‐8, and vascular endothelial growth factor in serum and vitreous fluid are increased in DM patients as compared to healthy people.^12^ However, the measurement of inflammatory cytokines is costly and not commonly used in daily clinical practice.

Also, routine blood biomarkers, like the complete blood counts, including counts of platelets, neutrophils, and lymphocytes, have been demonstrated in numerous DED studies to be useful in assessing inflammation levels. Ozarslan et al. studied several blood parameters that were compared between the healthy group and patients with DED; the results were statistically significant differences in levels of white blood cells and C‐reactive protein (CRP).^13^ As inflammation is also involved in the development of DM2, it is, therefore, likely that DED occurs more frequently in DM2 patients.^14^, ^15^ Interestingly, Alhalwani et al. noted a positive correlation between neutrophil‐to‐lymphocyte ratio (NLR) and CRP to albumin ratio (CAR) in DM2 patients with DED.^16^ Nevertheless, high NLR levels are also observed in other inflammatory conditions, including inflammatory bowel disease, other gastrointestinal conditions, and thyroiditis.^17^, ^18^, ^19^ Similarly, platelet‐to‐lymphocyte ratio (PLR) is increased in many conditions that are characterized by elevated inflammatory burden, such as thyroid conditions, gastrointestinal diseases, thyroiditis, irritable bowel disease, and COVID‐19 infection.^18^, ^20^, ^21^, ^22^, ^23^, ^24^

Two low‐cost indices, NLR and PLR, have been repeatedly assessed for their efficacy as diagnostic and prognostic tools for DED, DM, and other diseases. Raised levels of NLR and PLR have been associated with DED.^25^ The value of the systemic immune‐inflammation (SII) index has been used as a prognostic indicator for inflammatory diseases such as DM2.^26^ The combination of NLR, PLR, and SII measurements has proven more specific than testing for inflammatory biomarkers such as CRP.^27^ However, according to Meng et al., NLR, but not PLR, can be used to evaluate the severity of DED in diabetic patients. A recent study found a correlation between SII and type 2 diabetic retinopathy patients.^28^ Another study shows that SII level is increased among diabetes population.^29^ Therefore, this research aimed to elucidate further the association between systemic inflammatory biomarkers NLR, PLR, and SII (as immune response‐related indices) in DM2‐DED patients to improve our comprehension of DED's pathogenesis, treatment, and prevention.

## 2 METHODS

### 2.1 Study subject and sampling

**Figure 1:**
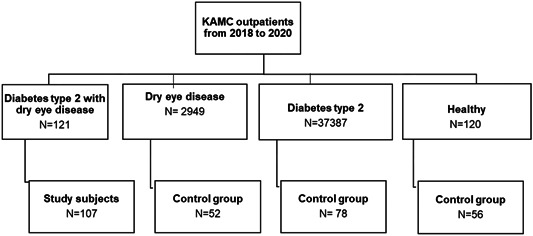
Flowchart of the collection of participants in the study. KAMC, King Abdulaziz Medical City.

For this research, a retrospective case‐control study included patients who had visited the King Abdulaziz Medical City (KAMC) outpatient clinic between January 2018 and December 2020. The information on healthy subjects was collected from the blood donation center. In total, 293 patients were included in this study; the study group was selected based on all patients with complete laboratory parameters DM2‐DED (*n* = 107). However, the control groups were selected randomly; DED only (*n* = 52), DM2 only (*n* = 78), and healthy (*n* = 56) groups (Figure 1). We found that the SII is an independent value that would be used as a predictor and prognostic marker in a group of patients with DM2‐DED compared to three control groups comprising patients with DED only, patients with DM2 only, and healthy subjects. Selection criteria for all participants included in the study criteria were that patients could be of either gender, must be 18 years of age or older, with eye diseases other than DED were excluded. Patients have either DM2, DED, or both, according to the group to which they would be allocated. Since the normal range for HbA1c is HbA1c ≤5.7%, subjects who were healthy or DED and had an HbA1c higher than 5.8% were excluded. Similarly, patients with diabetes with an HbA1c greater than 6.5%, including DM2 and DM2‐DED patients, were excluded if their HbA1c was less than 5.5%.

The participants in the study group who met these criteria were randomly selected for each group. Each of the four groups contained at least 50 subjects. The sample size of the study group was determined based on available D2M‐DED patients.

### 2.2 Sample size and data sheet

Data retrieved from patient files via the BESTCare 2.0A system were entered into Microsoft Excel. Sample sizes were calculated using an online clinical sample size calculator.^30^ Some 12,000 patients with DM2 and DED attended the KAMC outpatient clinic between January 2018 and December 2020. It was estimated that the required sample size should show a power of 80% and a significance of *p* = 0.05. The most recent prevalence of DED among patients with DM2 was estimated to be 54.3%.^31^ Accordingly, this case‐control study's minimum required sample size was 329 patients. This study had 107 individuals, the only DM2‐DED patients with complete data, and met the inclusion criteria.

The data collection sheet consisted of demographics (age and gender) and variables for the study and control groups, which ascertained the presence of DM2, other health problems, neutrophil count, lymphocyte count, platelet count, fasting blood glucose (FBS), and HbA1c levels. NLR, PLR, and SII indices were calculated and correlated with the presence of DED, DM2, DM2‐DED, or control subjects. NLR was computed by dividing the neutrophil count (×10^9^/L) by the lymphocyte count (×10^9^/L); PLR was computed by dividing the platelet count (×10^9^/L) by the lymphocyte count (×10^9^/L); and SII was computed by multiplying the platelet count (×10^9^/L) by NLR.^32^

### 2.3 Statistical analyses

All statistical analyses were carried out using Statistical Package for Social Science (SPSS) software, version 20.0 (IBM Corp.). For descriptive statistics, frequencies and percentages were computed for such categorical variables as gender. For inferential statistics, gender and condition frequencies were compared by application of the *χ* ^2^ test. The one‐way analysis of variance ANOVA test was used to compare ages among all groups and 95% confidence intervals (CIs) were calculated for all variables. While the laboratory findings data was confirmed to be nonparametric, this report presents the data using mean and standard deviation (mean ± SD) for better readability and ease of interpretation. However, recognizing the nonparametric nature of the data, the Kruskal−Wallis test was applied to assess statistical differences between groups based on median. Spearman's correlation test was used to assess the correlation among factors. A threshold for significance was set at *p* ≤ 0.05. The more information in supplamental materials as shown in Supporting Information S1: Table 1 & 3.

## 3 RESULTS

### 3.1 Baseline characteristics

The baseline characteristics of the study are presented in Table 1 (A and B). Table 1 (A) shows the gender results, and there was a statistically significant difference (*p* = 0.006) in gender distribution among all groups (DED: male 23.1%, female 76.9%; DM2: male 47.4%, female 52.6%; DM2‐DED: male 40.2%, female 59.6%, healthy: male 51.8%, female 48.2%). Table 1 (B) the age mean and SDs in years of all patients, showing a statistically significant difference arising between DED (48.7 ± 16.72 years), DM2 patients (59.1 ± 14.3 years), DM2‐DED patients (60.7 ± 11.85 years), and healthy subjects (53.9 ± 6.35 years) as evaluated by the post hoc Tukey HSD multiple‐comparison test (*p* < 0.001), more information in supplamental materials as shown in Supporting Information S1: Table 4.

**Table 1:** Baseline characteristics of the study and control groups by (A) gender and (B) age.

(A)			DED		DM2		DM2‐DED		Healthy		
		Total	*n* = 52	%	*n* = 78	%	*n* = 107	%	*n* = 56	%	*p*
Gender											
Male		121	12	(23.1)	37	(47.4)	43	(40.2)	29	(51.8)	0.012
Female		172	40	(76.9)	41	(52.6)	64	(59.8)	27	(48.2)	

(B)		Mean	SD	95% CI		*p*
Age	DED	48.7	16.72	44.02	53.33	<0.001
	DM2	59.1	14.30	55.83	62.28	
	DM2‐DED	60.7	11.85	58.48	63.02	
	Healthy	53.9	6.35	52.23	55.63	

*χ* ^2^ test (DED, dry eye disease; DM2, type‐2 diabetes; DM2‐DED, diabetes type‐2 with dry eye disease; healthy).

ANOVA (DED, dry eye disease; DM2, type‐2 diabetes; DM2‐DED, diabetes type‐2 with dry eye disease; healthy).

### 3.2 The characteristics of laboratory findings

In the DM2‐DED study group, the means ± SD of neutrophil, lymphocyte, and platelet counts were 4.5 ± 2.09 × 10^9^/L, 2.9 ± 1.35 × 10^9^/L, and 281.6 ± 93.36 × 10^9^/L, respectively; the mean ± SD of FBS and HbA1c levels were 9.67 ± 4.96 mmol/L and 8.7 ± 1.60% respectively; and the mean ± SD of PLR, NLR, and SII were 112.4 ± 57.29 × 10^9^/L, 1.9 ± 1.65 × 10^9^/L, and 545.0 ± 530.03 × 10^9^/L, respectively (Table 2).

**Table 2:** The characteristics of laboratory findings for the subjects of the study group (DM2‐DED) and control groups (DED, DM2, and healthy).

		Mean	SD	95% CI		*p*
Neutrophil	DED	3.4	1.68	2.91	3.85	<0.001
	DM2	4.3	2.00	3.90	4.80	
	DM2‐DED	4.5	2.09	4.05	4.86	
	Healthy	3.3	1.47	2.86	3.65	
Lymphocyte	DED	2.4	0.82	2.12	2.58	1.004
	DM2	2.8	1.03	2.58	3.05	
	DM2‐DED	2.9	1.35	2.66	3.18	
	Healthy	2.4	0.98	2.14	2.67	
Platelets	DED	274.4	89.71	249.43	299.38	1.461
	DM2	285.5	67.66	270.21	300.72	
	DM2‐DED	281.6	93.36	263.53	299.67	
	Healthy	264.1	64.56	246.85	281.43	
HbA1c	DED	5.3	0.34	5.25	5.44	<0.001
	DM2	9.2	7.90	7.45	11.01	
	DM2‐DED	8.7	1.60	8.41	9.03	
	Healthy	5.5	0.27	5.39	5.54	
FBS	DED	5.0	0.56	4.83	5.18	<0.001
	DM2	9.1	3.20	8.21	9.90	
	DM2‐DED	9.6	4.96	8.48	10.65	
	Healthy	5.4	1.07	5.16	5.73	
PLR	DED	129.7	60.29	112.94	146.51	0.291
	DM2	117.6	63.09	103.31	131.94	
	DM2‐DED	112.4	57.29	101.29	123.46	
	Healthy	125.6	55.87	110.68	140.60	
NLR	DED	1.7	1.51	1.27	2.11	0.299
	DM2	2.0	1.93	1.54	2.42	
	DM2‐DED	1.9	1.65	1.59	2.23	
	Healthy	1.5	0.81	1.28	1.72	
SII	DED	461.0	515.48	317.52	604.54	0.271
	DM2	537.9	539.27	415.53	660.32	
	DM2‐DED	545.4	530.03	442.87	648.02	
	Healthy	401.9	267.04	330.38	473.41	

ANOVA (DED, dry eye disease; FBS, fasting blood sugar; HbA1c, glycosylated hemoglobin; NLRs, neutrophil‐to‐lymphocyte ratios; PLRs, platelet‐to‐lymphocyte ratios; SII, systemic immune‐inflammation index).

In the comparison between all groups, there was a statistically significant difference increase in levels of neutrophils, HbA1c, and FBS (*p* < 0.001, *p* < 0.001, *p* < 0.001, *p* < 0.001, and *p* = 0.05, respectively). The mean values for lymphocytes, platelets, PLR, NLR, and SII among all groups diverged widely, although the differences were not a statistically significant difference (*p* = 1.004, *p* = 1.461, *p* = 0.291, *p* = 0.299, and *p* = 0.271, respectively). This finding showed that the SII was a better‐differentiating index among all groups than PLR and NLR. Both groups (DM2 and DM2‐DED) have higher SII values (537.9 and 545.4, respectively) and HbA1c levels (9.2 ± 7.90% and 8.7 ± 1.60%, respectively) as compared to the DED and healthy groups.

### 3.3 Correlation study

Correlation analysis for each group of the HbA1c against the PLRs, NLRs, and SII is shown in Figure 2. In the DM2‐DED group, there were positively correlated results between the HbA1c versus each of the PLR, NLR, and SII variables with no staticly significant diffrences (*p* = 0.705, *p* = 0.754, and *p* = 0.501, respectively), more information in supplamental materials as shown in Supporting Information S1: Table 2.

Figure 2Correlation between HbA1c and PLR (A), HbA1c and NLR (B), and HbA1c and SII (C) for the study and control groups. HbA1c, hemoglobin A1c; NLR, neutrophil‐to‐lymphocyte ratio; PLR, platelet‐to‐lymphocyte ratio; SII, systemic immune‐inflammation index.

**Figure:**
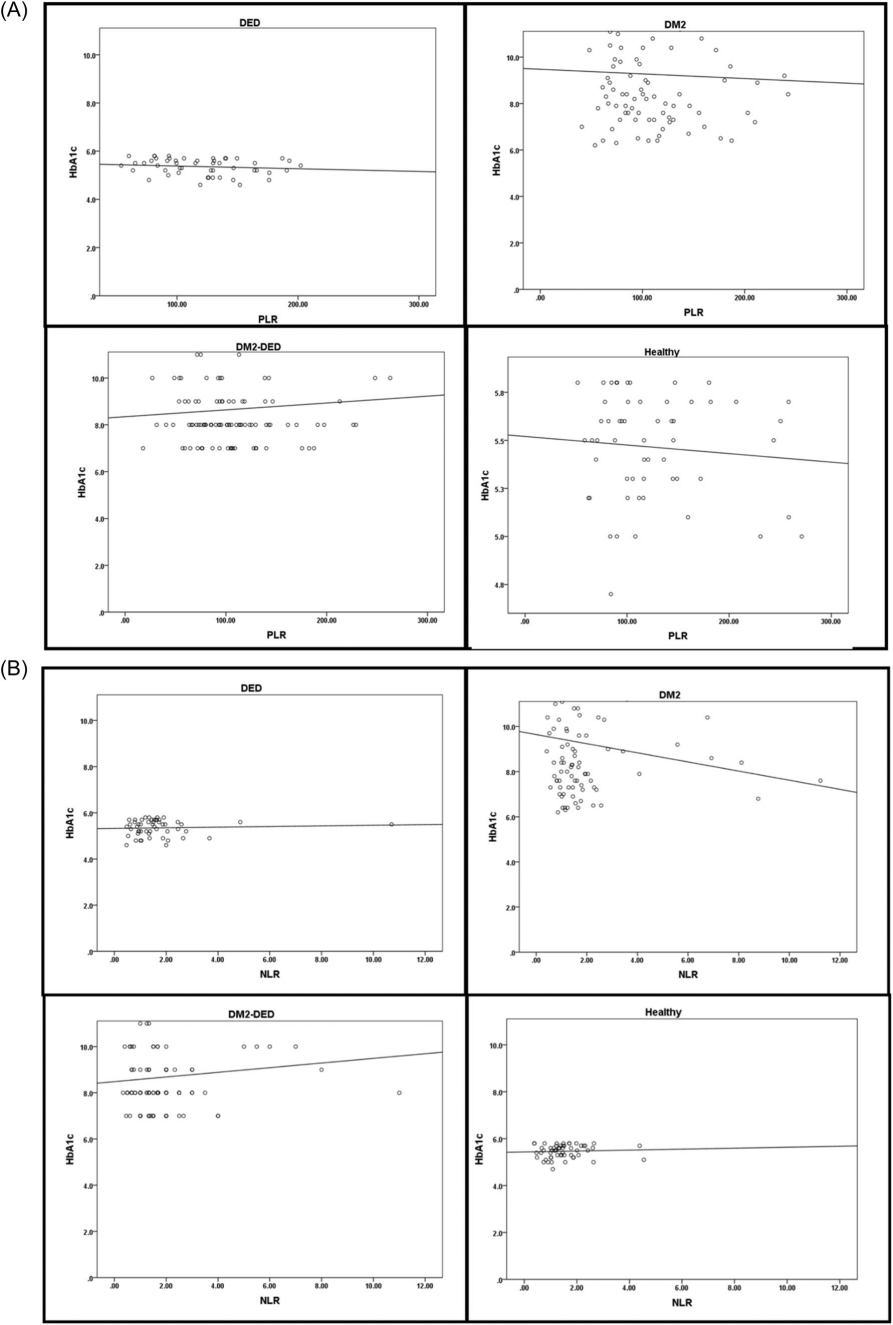

**Figure:**
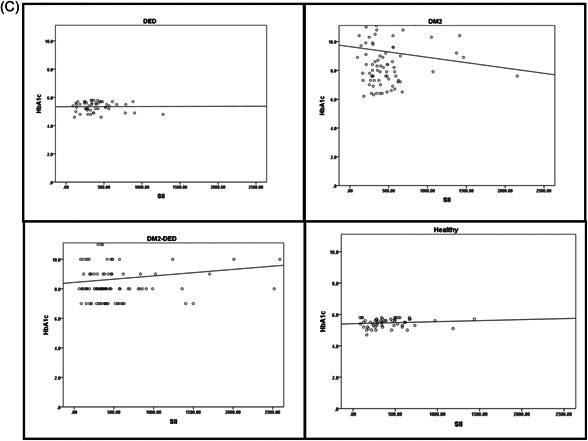

## 4 DISCUSSION

To our knowledge, this research revealed the first study evaluating the SII levels in patients with DM2‐DED compared to DED, DM2, and healthy controls. Although the dey eye disease progression and risk factors of type 2 diabetes patients have not been fully understood.

The subjects of this study were healthy patients with type 2 diabetes and DED with mean ages in the elderly range, with females being older than males. Our results are consistent with other research that has demonstrated that age and gender are significant risk factors for both type 2 diabetes and DED patients' outcomes.^33^, ^34^, ^35^

Because both PLR and NLR have previously been used as markers for inflammation.^36^, ^37^ Also, PLR, NLR, and SII are standard tests that are inexpensive and predictive indicators for diabetic patients.^38^, ^39^, ^40^, ^41^ As well as in diabetes, PLR, NLR, and SII levels are considered predictive and prognostic markers for metabolic diseases such as diabetes with complications.^40^, ^41^, ^42^, ^43^ Based on that, this study employed PLR, NLR, and SII laboratory findings for patients with DM2‐DED, alongside, although few studies have been carried out to investigate the association of DM2 with DED patients, further investigation is required.^4^, ^16^, ^31^, ^33^, ^44^, ^45^

The inflammatory markers of PLR, NLR, and SII values were detected and compared across all groups. Our finding of the PLR, NLR, and SII mean values of the healthy subjects is compatible with previous studies for healthy subject values of PLR, NLR, and SII, as reported in the previous research, specifically 117.05 ± 47.73, 1.70 ± 0.70, and 569.73 ± 326.67, respectively.^46^, ^47^

In the DM2‐DED study group, the means of PLR and NLR levels were higher than the healthy control group. However, there was no significant difference in the NLR, PLR, and SII of individuals between all groups, so we assume that this did not affect the study's outcome. This result aligns with several studies for DED patients only^48^, ^49^ and for DM2 patients only.^50^, ^51^, ^52^, ^53^

Studies related to DED patients only, the study findings reported by Meng et al., which showed that PLR (117.48 ± 54.68) and NLR (2.59 ± 1.25) in the DED group were higher than the healthy control group PLR (115.48 ± 54.33) and NLR (2.20 ± 1.24) values.^25^ Another study also reported the same of DED patients and showed that the mean PLR, NLR, and SII levels were significantly higher in DED patients than in the healthy control group.^13^ Additionally, a previous study shows that DED patients have higher NLR and PLR values (2.6 ± 1.2 and 138.4 ± 62.6) than are associated with healthy NLR and PLR values (1.84 ± 0.5 and 118.5 ± 64.7).^48^

Looking at studies related to DM2 patients only, a study conducted by Dik et al. found that NLR and PLR values (145.9 and 2.4, respectively) were increased in DM patients compared to NLR and PLR values (2.1 and 146.7, respectively) for the healthy control group.^54^ Moreover, Nagabhushan et al. found that NLR and PLR values in DM2 patients (2.5 and 119.7, respectively) were increased in DM patients with poorly controlled DM2 as compared with NLR and PLR values (1.02 and 95.2, respectively) for the healthy control group.^55^ A similar study by Mourcy et al. found that differences in NLR values were statistically significant between DM2, DM2 with retinopathy, and healthy groups (1.41, 2.11, and 1.40), respectively.^56^

Interestingly, the DM2‐DED group outperforms the healthy group in NLR but not PLR. This outcome is consistent with a study that Meng et al. published, which indicated that the NLR, but not the PLR, of DED patients was reportedly higher than that of healthy subjects.^25^ Also, NLR and PLR value are dependent on population characteristics such as race.^38^, ^57^

The DM2‐DED had increased levels of SII as well as HbA1c. In addition, SII values were found to be considerably higher in patients with DM2‐DED and DM2 than healthy and DED only. This finding aligns with many studies on chronic diseases compared with healthy subjects. A study reported that PLR, NLR, and SII levels were higher than the healthy control group in numerous earlier investigations on inflammatory diseases, including hepatocellular carcinoma and diabetic depression.^26^ The relationships between outpatient PLR, NLR, and SII levels with blood glucose regulation have previously been studied by several diabetes groups, though not the DM2‐DED group.^58^, ^59^, ^60^

Also, all groups' SII results were higher than those of the healthy group, consistent with other research linking elevated SII levels to inflammatory diseases such as diabetes only, and DED only.^13^, ^42^, ^47^, ^58^ In the study by Ozarslan Ozcan et al., DED patients showed significantly higher SII, PLR, and NLR levels than the healthy group. Despite the discrepancy, our study verified the findings of Ozarslan Ozcan et al., which showed a positive correlation between SII, the NLR, and PLR biomarkers.

According to Atak et al.'s study of DM2 patients, a positive correlation exists between PLR and HbA1c levels, supporting our findings that PLR levels are positively correlated with HbA1c levels in the DM2‐DED group.^22^

Additionally, a diabetic study has examined the links between patients' NLR levels and those of blood glucose, and their findings are consistent with our findings in DM2‐DED patients.^61^

Hyperglycemia is related to the elevated production of reactive oxygen species from neutrophils, which in turn reflects alterations to neutrophil clearance and function and prolonged inflammation.^61^ The elevated SII was supported by poor HbA1c levels and recurrence in patients with DM2 and DM2‐DED. Additionally, recently published work showed a relationship between HbA1c and SII (*r*'s = 0.145, *p* = 0.01). In contrast, SII supersedes NLR and PLR as a prognostic factor for DM2‐DED outcomes. The results were satisfactory and suggested that DM2 patients with poor glycaemic control demonstrated glucose levels that made them susceptible to DED.^5^

DM2 patients with poor HbA1c levels had more risk factors for DED.^31^ This study supported our findings that SII values were higher in those patients with DM2 and DM2‐DED, who are characterized by poor HbA1c levels compared to healthy and DED groups.

In light of the correlation findings, a combination of both SII and HbA1c may be suitable biomarker for the pathogenesis and prediction of the disease of DM2‐DED. This study adds new information to understanding the role of inflammation in the pathophysiology of DM2‐DED. A potential explanation of a better prognostic value might be that SII was more comprehensive in reflecting the status of inflammatory and immune responses than the other established factors.

## 5 CONCLUSION

In conclusion, this study supports the idea that inflammation may play an essential role in the development of diabetic patients presenting with DED. The results of our study indicate that SII levels are differences between all groups DM2‐DED, DED, DM2, and healthy subjects. Additionally, SII has higher value compared to NLR and PLR among all groups, which consider an inflammatory indicator. There is a positive correlation between HbA1c levels and SII in the DM2‐DED group than in other groups. Moreover, our study demonstrated that higher SII may be a potential marker for DM2‐DED development. These findings, however, indicate that additional investigation is required to discover other potential biomarkers that can be used to identify and prevent the early stages of DED in DM2 patients.

## 6 LIMITATIONS

The ability of this investigation to evaluate the severity of DED is constrained by the absence of relevant retrospective data. However, additional prospective, randomized controlled studies involving a larger patient population are required to generate more robust data.

## AUTHOR CONTRIBUTIONS

**Amani Y. Alhalwani**: Conceptualization; investigation; project administration; supervision; validation; writing—original draft; writing—review and editing. **Shatha Jambi**: Data curation; formal analysis; methodology; software; validation; visualization; writing—review and editing. **Anwar Borai**: Resources; writing—review and editing. **Muhammad Anwar Khan**: Formal analysis; writing—review and editing. **Hashem Almarzouki**: Resources. **Mohieldin Elsayid**: Resources. **Abdullah Fahad Aseri**: Data curation; writing—review and editing. **Nada O. Taher**: Data curation; writing—review and editing. **Ali Alghamdi**: Data curation; writing—review and editing. **Abdulwahab Alshehri**: Data curation; writing—review and editing. All authors have read and approved the final version of the manuscript.

## CONFLICT OF INTEREST STATEMENT

The authors declare no conflict of interest.

## ETHICS STATEMENT

The study obtained ethical approval from the Institutional Review Board at King Abdullah International Medical Research Centre (Jeddah, Saudi Arabia) with the protocol number RSS21J‐002‐06. As the study was retrospective, it could be conducted without patients' informed consent. Patient data were chosen randomly.

### TRANSPARENCY STATEMENT

The lead author Amani Y. Alhalwani affirms that this manuscript is an honest, accurate, and transparent account of the study being reported; that no important aspects of the study have been omitted; and that any discrepancies from the study as planned (and, if relevant, registered) have been explained.

## Supporting information

Supporting information.

## Data Availability

Research data are not shared. The data presented in this study are available on request from the corresponding author. The corresponding had full access to all of the data in this study and take complete responsibility for the data's integrity and the data analysis's accuracy.
