# Supplementary material for: Assessment of the systemic immune‐inflammation index in type 2 diabetic patients with and without dry eye disease: A case‐control study
Source: Health Sci Rep. 2024 May 1;7(5):e1954. doi: 10.1002/hsr2.1954 (PMC11063262; doi:10.1002/hsr2.1954)
Supplement: Supplementary file 1 — Supporting information. [file HSR2-7-e1954-s001.pdf]

Electronic Supplementary Material

Assessment of the Systemic Immune-Inflammation Index in Type 2 Diabetic Patients with and without Dry Eye Disease: A Case-Control study.

Amani Y. Alhalwani[1,2*], Shatha Jambi[1,2], Anwar Borai[1,2,3], Muhammad Anwar Khan[1,2], Hashem Almarzouki[1,2,3], Mohieldin Elsayid[1,2,], Abdullah Fahad Aseri[4], Nada O. Taher[1], Ali Alghamdi[4], Abdulwahab Alshehri[5]

Table S1: Laboratory findings data using non-parametric analysis.

Type		Mean Rank	Median	IQR	p
**Neutrophil**	DED	115.6	2.9	2.1-4.3	<0.001
	DM2	162.2	4.1	3-5.3	
	DM2-DED	165.1	4	3-5	
	Healthy	113.6	3	2-4.4	
**Lymphocyte**	DED	123.3	2.3	1.9-2.7	0.004
	DM2	161.0	2.8	2.2-3.6	
	DM2-DED	158.3	3	2-3	
	Healthy	120.9	2.3	1.73	
**Platelets**	DED	139.8	271	216.5-307.5	0.456
	DM2	157.5	276	242-330.3	
	DM2-DED	146.0	273	224-322	
	Healthy	135.6	272	215-298	
**HbA1c**	DED	49.2	5.5	5.1-5.6	<0.001
	DM2	194.5	8.1	7.3-9.3	
	DM2-DED	205.8	8	8-10	
	Healthy	59.4	5.5	5.3-5.7	
**PLR**	DED	166.7	125.8	92.2-148.9	0.081
	DM2	138.6	100.4	77-133.3	
	DM2-DED	134.2	103	76.3-129.8	
	Healthy	156.6	110	85.6-146.6	
**NLR**	DED	141.0	1.4	0.9-1.9	0.742
	DM2	147.5	1.4	1-2	
	DM2-DED	150.9	1.5	1-2	
	Healthy	136.8	1.4	1-1.8	
**SII**	DED	135.0	351.4	259.3-508.5	0.359
	DM2	154.5	389	291.3-564.2	
	DM2-DED	150.5	396.7	254.7-584.6	
	Healthy	133.6	342.9	236.6-531.4	
**FBS**	DED	50.0	4.9	4.7-5.4	<0.001
	DM2	164.2	8.3	7-10.1	
	DM2-DED	154.8	8	6-12	
	Healthy	71.8	5.3	4.9-5.9	

Kruskal Wallis test

Table S2: Spearman's correlations for all parameters in (A) DED, (B) DM2, (C) DED, and (D) Healthy groups.

**(A)**	Spearman's rho		HbA1c	Neutrophil	PLR	SII_3	Lymphocyte	Platelets	FBS
DED	Neutrophil	Correlation Coefficient	.283*						
		Sig. (2-tailed)	.042						
	PLR	Correlation Coefficient	-.243	-.151					
		Sig. (2-tailed)	.082	.287					
	SII_3	Correlation Coefficient	.057	.755**	.466**				
		Sig. (2-tailed)	.690	.000	.001				
	Lymphocyte	Correlation Coefficient	.348*	.264	-.745**	-.225			
		Sig. (2-tailed)	.012	.058	.000	.109			
	Platelets	Correlation Coefficient	.056	.231	.381**	.423**	.246		
		Sig. (2-tailed)	.693	.100	.005	.002	.079		
	FBS	Correlation Coefficient	.172	.128	-.091	-.028	-.051	-.046	
		Sig. (2-tailed)	.283	.425	.573	.860	.752	.776	
	NLR	Correlation Coefficient	.066	.635**	.414**	.798**	-.473**	-.080	.026
		Sig. (2-tailed)	.644	.000	.002	.000	.000	.571	.870

**(B)**	Spearman's rho		HbA1c	Neutrophil	PLR	SII_3	Lymphocyte	Platelets	FBS
DM2	Neutrophil	Correlation Coefficient	.054						
		Sig. (2-tailed)	.640						
	PLR	Correlation Coefficient	-.107	-.012					
		Sig. (2-tailed)	.354	.915					
	SII_3	Correlation Coefficient	-.057	.622**	.720**				
		Sig. (2-tailed)	.623	.000	.000				
	Lymphocyte	Correlation Coefficient	.027	-.005	-.814**	-.595**			
		Sig. (2-tailed)	.819	.967	.000	.000			
	Platelets	Correlation Coefficient	-.094	-.066	.387**	.218	.151		
		Sig. (2-tailed)	.414	.568	.000	.056	.190		
	FBS	Correlation Coefficient	.393**	.130	.023	.089	-.085	-.054	
		Sig. (2-tailed)	.002	.330	.861	.508	.527	.690	
	NLR	Correlation Coefficient	-.034	.704**	.524**	.901**	-.645**	-.154	.078
		Sig. (2-tailed)	.770	.000	.000	.000	.000	.182	.559

**(C)** Spearman's rho			HbA1c	Neutrophil	PLR	SII_3	Lymphocyte	Platelets	FBS
DM2-DED	Neutrophil	Correlation Coefficient	.078						
		Sig. (2-tailed)	.428						
	PLR	Correlation Coefficient	.037	.288**					
		Sig. (2-tailed)	.705	.003					
	SII_3	Correlation Coefficient	.066	.825**	.721**				
		Sig. (2-tailed)	.501	.000	.000				
	Lymphocyte	Correlation Coefficient	.055	-.032	-.732**	-.427**			
		Sig. (2-tailed)	.574	.746	.000	.000			
	Platelets	Correlation Coefficient	.078	.279**	.406**	.400**	.231*		
		Sig. (2-tailed)	.431	.004	.000	.000	.018		
	FBS	Correlation Coefficient	.445**	-.020	.100	.058	-.029	.059	
		Sig. (2-tailed)	.000	.861	.369	.602	.794	.593	
	NLR	Correlation Coefficient	.031	.769**	.645**	.919**	-.599**	.072	.025
		Sig. (2-tailed)	.754	.000	.000	.000	.000	.468	.821

**(D)** Spearman's rho			HbA1c	Neutrophil	PLR	SII_3	Lymphocyte	Platelets	FBS
Healthy	Neutrophil	Correlation Coefficient	.212						
		Sig. (2-tailed)	.116						
	PLR	Correlation Coefficient	-.057	.033					
		Sig. (2-tailed)	.677	.810					
	SII_3	Correlation Coefficient	.164	.769**	.614**				
		Sig. (2-tailed)	.227	.000	.000				
	Lymphocyte	Correlation Coefficient	.148	.315*	-.757**	-.223			
		Sig. (2-tailed)	.277	.018	.000	.098			
	Platelets	Correlation Coefficient	.161	.546**	.319*	.613**	.301*		
		Sig. (2-tailed)	.237	.000	.017	.000	.024		
	FBS	Correlation Coefficient	.216	.266*	.010	.186	-.027	.012	
		Sig. (2-tailed)	.110	.047	.942	.171	.846	.930	
	NLR	Correlation Coefficient	.143	.659**	.625**	.918**	-.440**	.301*	.164
		Sig. (2-tailed)	.292	.000	.000	.000	.001	.024	.227

Table S3: Post hoc analysis for age

(I) Type		Mean Difference (I-J)	Std. Error	p-value	95% Confidence Interval	
					Lower Bound	Upper Bound
DED	DM2	-10.378*	2.286	.000	-16.29	-4.47
	DM2-DED	-12.075*	2.159	.000	-17.65	-6.50
	Healthy	-5.255	2.459	.144	-11.61	1.10
DM2	DED	10.378*	2.286	.000	4.47	16.29
	DM2-DED	-1.696	1.901	.809	-6.61	3.22
	Healthy	5.123	2.237	.103	-.66	10.90
DM2-DED	DED	12.075*	2.159	.000	6.50	17.65
	DM2	1.696	1.901	.809	-3.22	6.61
	Healthy	6.819*	2.106	.007	1.38	12.26
Healthy	DED	5.255	2.459	.144	-1.10	11.61
	DM2	-5.123	2.237	.103	-10.90	.66
	DM2-DED	-6.819*	2.106	.007	-12.26	-1.38

Table S4: Post hoc analysis for all laboratory tests

Dependent Variable			Mean Difference (I-J)	Std. Error	p-value	95% Confidence Interval	
						Lower Bound	Upper Bound
Neutrophil	DED	DM2	-.96814*	.33916	.024	-1.8446	-.0917
		DM2-DED	-1.07541*	.32041	.005	-1.9034	-.2474
		Healthy	.12798	.36389	.985	-.8124	1.0683
	DM2	DED	.96814*	.33916	.024	.0917	1.8446
		DM2-DED	-.10727	.28350	.982	-.8399	.6253
		Healthy	1.09612*	.33185	.006	.2386	1.9537
	DM2-DED	DED	1.07541*	.32041	.005	.2474	1.9034
		DM2	.10727	.28350	.982	-.6253	.8399
		Healthy	1.20339*	.31266	.001	.3954	2.0114
	Healthy	DED	-.12798	.36389	.985	-1.0683	.8124
		DM2	-1.09612*	.33185	.006	-1.9537	-.2386
		DM2-DED	-1.20339*	.31266	.001	-2.0114	-.3954
Lymphocyte	DED	DM2	-.46484	.20096	.097	-.9841	.0545
		DM2-DED	-.56502*	.18985	.017	-1.0556	-.0744
		Healthy	-.05570	.21561	.994	-.6129	.5015
	DM2	DED	.46484	.20096	.097	-.0545	.9841
		DM2-DED	-.10018	.16798	.933	-.5343	.3339
		Healthy	.40914	.19663	.162	-.0990	.9173
	DM2-DED	DED	.56502*	.18985	.017	.0744	1.0556
		DM2	.10018	.16798	.933	-.3339	.5343
		Healthy	.50932*	.18526	.032	.0306	.9881
	Healthy	DED	.05570	.21561	.994	-.5015	.6129
		DM2	-.40914	.19663	.162	-.9173	.0990
		DM2-DED	-.50932*	.18526	.032	-.9881	-.0306
Platelets	DED	DM2	-11.058	14.562	.873	-48.69	26.57
		DM2-DED	-7.196	13.792	.954	-42.84	28.45
		Healthy	10.261	15.664	.914	-30.22	50.74
	DM2	DED	11.058	14.562	.873	-26.57	48.69
		DM2-DED	3.862	12.158	.989	-27.56	35.28
		Healthy	21.319	14.246	.441	-15.50	58.13

	DM2-DED	DED	7.196	13.792	.954	-28.45	42.84
		DM2	-3.862	12.158	.989	-35.28	27.56
		Healthy	17.457	13.459	.566	-17.32	52.24
	Healthy	DED	-10.261	15.664	.914	-50.74	30.22
		DM2	-21.319	14.246	.441	-58.13	15.50
		DM2-DED	-17.457	13.459	.566	-52.24	17.32
HbA1c	DED	DM2	-3.8788*	.7511	.000	-5.820	-1.938
		DM2-DED	-3.3715*	.7092	.000	-5.204	-1.539
		Healthy	-.1162	.8079	.999	-2.204	1.972
	DM2	DED	3.8788*	.7511	.000	1.938	5.820
		DM2-DED	.5073	.6246	.849	-1.107	2.121
		Healthy	3.7626*	.7348	.000	1.864	5.661
	DM2-DED	DED	3.3715*	.7092	.000	1.539	5.204
		DM2	-.5073	.6246	.849	-2.121	1.107
		Healthy	3.2553*	.6919	.000	1.467	5.043
	Healthy	DED	.1162	.8079	.999	-1.972	2.204
		DM2	-3.7626*	.7348	.000	-5.661	-1.864
		DM2-DED	-3.2553*	.6919	.000	-5.043	-1.467
FBS	DED	DM2	-4.0503*	.6895	.000	-5.834	-2.266
		DM2-DED	-4.5614*	.6451	.000	-6.231	-2.892
		Healthy	-.4362	.6946	.923	-2.233	1.361
	DM2	DED	4.0503*	.6895	.000	2.266	5.834
		DM2-DED	-.5111	.5783	.813	-2.008	.985
		Healthy	3.6141*	.6331	.000	1.976	5.252
	DM2-DED	DED	4.5614*	.6451	.000	2.892	6.231
		DM2	.5111	.5783	.813	-.985	2.008
		Healthy	4.1252*	.5844	.000	2.613	5.637
	Healthy	DED	.4362	.6946	.923	-1.361	2.233
		DM2	-3.6141*	.6331	.000	-5.252	-1.976
		DM2-DED	-4.1252*	.5844	.000	-5.637	-2.613
PLR	DED	DM2	12.09910	10.61804	.665	-15.3400	39.5382
		DM2-DED	17.34750	10.03113	.310	-8.5749	43.2699
		Healthy	4.08319	11.39233	.984	-25.3569	33.5232
	DM2	DED	-12.09910	10.61804	.665	-39.5382	15.3400

		DM2-DED	5.24840	8.87548	.935	-17.6876	28.1844
		Healthy	-8.01591	10.38922	.867	-34.8637	18.8319
	DM2-DED	DED	-17.34750	10.03113	.310	-43.2699	8.5749
		DM2	-5.24840	8.87548	.935	-28.1844	17.6876
		Healthy	-13.26431	9.78860	.529	-38.5600	12.0314
	Healthy	DED	-4.08319	11.39233	.984	-33.5232	25.3569
		DM2	8.01591	10.38922	.867	-18.8319	34.8637
		DM2-DED	13.26431	9.78860	.529	-12.0314	38.5600
NLR	DED	DM2	-.28799	.28484	.743	-1.0241	.4481
		DM2-DED	-.21516	.26910	.855	-.9106	.4802
		Healthy	.19113	.30561	.924	-.5986	.9809
	DM2	DED	.28799	.28484	.743	-.4481	1.0241
		DM2-DED	.07283	.23810	.990	-.5425	.6881
		Healthy	.47912	.27870	.316	-.2411	1.1993
	DM2-DED	DED	.21516	.26910	.855	-.4802	.9106
		DM2	-.07283	.23810	.990	-.6881	.5425
		Healthy	.40629	.26259	.411	-.2723	1.0849
	Healthy	DED	-.19113	.30561	.924	-.9809	.5986
		DM2	-.47912	.27870	.316	-1.1993	.2411
		DM2-DED	-.40629	.26259	.411	-1.0849	.2723
SII	DED	DM2	-76.89500	88.03096	.819	-304.3845	150.5945
		DM2-DED	-84.41039	83.16510	.741	-299.3256	130.5048
		Healthy	59.13122	94.45040	.924	-184.9474	303.2098
	DM2	DED	76.89500	88.03096	.819	-150.5945	304.3845
		DM2-DED	-7.51540	73.58398	1.000	-197.6710	182.6403
		Healthy	136.02622	86.13389	.392	-86.5609	358.6133
	DM2-DED	DED	84.41039	83.16510	.741	-130.5048	299.3256
		DM2	7.51540	73.58398	1.000	-182.6403	197.6710
		Healthy	143.54161	81.15436	.291	-66.1774	353.2606
	Healthy	DED	-59.13122	94.45040	.924	-303.2098	184.9474

	DM2	-136.02622	86.13389	.392	-358.6133	86.5609
	DM2-DED	-143.54161	81.15436	.291	-353.2606	66.1774

Figure S1: Post hoc Dunn test for **(A; Neutrophils) (B; Lymphocytes), (C; HbA1c), and (D; FBS).**

(A) Post hoc Dunn test for Neutrophil

Each node shows the sample average rank of Type.

Sample1-Sample2	Test Statistic	Std. Error	Std. Test Statistic	Sig.	Adj.Sig.
Healthy-DED	1.998	16.135	.124	.901	1.000
Healthy-DM2	48.590	14.714	3.302	.001	.006
Healthy-DM2-DED	51.488	13.864	3.714	.000	.001
DED-DM2	-46.592	15.039	-3.098	.002	.012
DED-DM2-DED	-49.490	14.207	-3.483	.000	.003
DM2-DM2-DED	-2.897	12.571	-.230	.818	1.000

Each row tests the null hypothesis that the Sample 1 and Sample 2 distributions are the same.
Asymptotic significances (2-sided tests) are displayed. The significance level is .05.

(B) Post hoc Dunn test for Lymphocyte

Each node shows the sample average rank of Type.

Sample1-Sample2	Test Statistic	Std. Error	Std. Test Statistic	Sig.	Adj.Sig.
Healthy-DED	2.444	16.114	.152	.879	1.000
Healthy-DM2-DED	37.374	13.845	2.699	.007	.042
Healthy-DM2	40.062	14.695	2.726	.006	.038
DED-DM2-DED	-34.930	14.189	-2.462	.014	.083
DED-DM2	-37.618	15.019	-2.505	.012	.074
DM2-DED-DM2	2.688	12.554	.214	.830	1.000

Each row tests the null hypothesis that the Sample 1 and Sample 2 distributions are the same.
Asymptotic significances (2-sided tests) are displayed. The significance level is .05.

(C) Post hoc Dunn test for HbA1c

Each node shows the sample average rank of Type.

Sample1-Sample2	Test Statistic	Std. Error	Std. Test Statistic	Sig.	Adj.Sig.
DED-Healthy	-10.273	16.276	-.631	.528	1.000
DED-DM2	-145.288	15.130	-9.602	.000	.000
DED-DM2-DED	-156.593	14.287	-10.961	.000	.000
Healthy-DM2	135.015	14.803	9.121	.000	.000
Healthy-DM2-DED	146.320	13.939	10.497	.000	.000
DM2-DM2-DED	-11.305	12.583	-.898	.369	1.000

Each row tests the null hypothesis that the Sample 1 and Sample 2 distributions are the same.
Asymptotic significances (2-sided tests) are displayed. The significance level is .05.

(D) Post hoc Dunn test for FBS

Each node shows the sample average rank of Type.

Sample1-Sample2	Test Statistic	Std. Error	Std. Test Statistic	Sig.	Adj.Sig.
DED-Healthy	-21.870	14.145	-1.546	.122	.732
DED-DM2-DED	-104.832	13.137	-7.980	.000	.000
DED-DM2	-114.256	14.041	-8.137	.000	.000
Healthy-DM2-DED	82.962	11.901	6.971	.000	.000
Healthy-DM2	92.385	12.893	7.166	.000	.000
DM2-DED-DM2	9.424	11.778	.800	.424	1.000

Each row tests the null hypothesis that the Sample 1 and Sample 2 distributions are the same.
Asymptotic significances (2-sided tests) are displayed. The significance level is .05.
